# Educational Concerns, Health Concerns and Mental Health During Early COVID-19 School Closures: The Role of Perceived Support by Teachers, Family, and Friends

**DOI:** 10.3389/fpsyg.2021.733683

**Published:** 2022-01-25

**Authors:** Lena Dändliker, Isabel Brünecke, Paola Citterio, Fabienne Lochmatter, Marlis Buchmann, Jeanine Grütter

**Affiliations:** ^1^Jacobs Center for Productive Youth Development, University of Zurich, Zurich, Switzerland; ^2^Department of Psychology, University of Konstanz, Konstanz, Germany; ^3^Empirical Education Research, University of Konstanz, Konstanz, Germany

**Keywords:** educational concerns, perceived friend support, perceived family support, perceived teacher support, mental health, latent profiles, COVID-19 school closures, health concerns

## Abstract

This study investigated whether school closures and health-related uncertainties in the early phases of the COVID-19 pandemic posed risk factors for adolescents’ mental health and whether perceived social support by parents, teachers, and friends functioned as protective factors. In particular, we argued that perceived social support would buffer negative associations between educational and health concerns and mental health. Based on a person-centered approach, we first examined resilience profiles. These profiles reflect configurations regarding the levels of these risk and protective factors and levels of mental health. Second, we analyzed whether these risk and protective factors predicted adolescents’ mental health differently by using a variable-centered approach. The sample consisted of 1’562 adolescents (*M*age = 16.18, *SD* = 1.48, range = 14-20 years; 72% females) in lower and higher secondary education from three regions: German-speaking part of Switzerland, *N* = 486; Italian-speaking part of Switzerland, *N* = 760; and Northern Italy *N* = 316. Results from the person-centered approach revealed three latent profiles characterized by low (19%), average (47%), or high resilience (34%). Lower resilience was associated with higher educational concerns, lower perceived social support, and lower mental health, while high resilience was characterized by lower concerns, higher support, and higher mental health. Importantly, educational concerns varied more between profiles than health concerns, and perceived teacher and family support varied more than perceived friend support. Corroborating these findings, the variable-centered approach (i.e., a path analysis) revealed that educational concerns were a stronger predictor than health concerns and pointed to a higher relative importance of perceived family support for adolescents’ mental health relative to perceived teacher and friend support. Taken together, the findings suggest that adolescents’ educational concerns and perceived family support, respectively, were stronger risk and protective factors for their mental health during school closures related to the COVID-19 pandemic. Finally, adolescents from regions being more exposed to the COVID-19 pandemic, namely, Italian-speaking part of Switzerland and Northern Italy, were more likely classified in the low or the average rather than in the high resilience profile compared to students from the region with lower exposure, that is, the German-speaking part of Switzerland.

## Introduction

In early 2020, the spread of the coronavirus SARS-CoV-2 led severely affected countries like Switzerland and Italy to take protective and containment measures, such as school closures and the conversion from in-school to distance learning. Due to these changes, adolescents faced new challenges in their educational environment, such as increased demand for self-centered learning, insecurities about their future and fear of grade retention, in particular (Klootwijk et al., 2021). At the same time, face-to-face support from teachers was restricted in certain schools. Teacher support was particularly limited during the lockdown, as many teachers had to adjust to their new roles, including the application of new teaching technologies (Beteille et al., 2020; Korzycka et al., 2021). In addition to these educational concerns, adolescents encountered uncertainties about the virus and fear about infection (Brooks et al., 2020). This occurred at a time when they were not able to interact in person with their friends, who could be an important source of support in times of crisis; hence, restricted social support may have exacerbated psychosocial and internalizing problems (Bernasco et al., 2021). Evidence shows that such pandemic-related stressors have been negatively associated with adolescents’ mental health (Guessoum et al., 2020; Shanahan et al., 2020).

While potential risks for adolescents’ mental health during the crisis have been documented (e.g., Shanahan et al., 2020), little is known about the relative role of educational and health-related risk factors, reflecting individual stressors for adolescents. More research is also needed to better understand how adolescents perceived different sources of social support and how these sources were related to their mental health during pandemic-related school closures. While the positive role of perceived social support from teachers, families and friends for adolescents’ mental health has been well documented in general (Armstrong et al., 2005; Pinkerton and Dolan, 2007; Suldo et al., 2009; Traylor et al., 2016), first evidence points to its significant role during the COVID-19 pandemic (Ellis et al., 2020; Bernasco et al., 2021; Klootwijk et al., 2021; Ye et al., 2021). The current research aimed to build on these first insights by studying the relative role of these support systems during school closures in early phases of the pandemic. Due to the new situation, the relative role of particular social support systems may have changed, as support from face-to-face interactions with teachers and friends were less likely, while family interaction patterns have been more frequent at the same time (Ellis et al., 2020; Fegert et al., 2020; Prime et al., 2020). Therefore, perceived family support may have gained in significance for adolescents’ mental health, among other things, also in their supporting role regarding distance learning (Garrote et al., 2021).

Based on a resilience framework, the current study investigated adolescents’ mental health during school closures at the onset of the COVID-19 pandemic taken as an indicator for adolescents’ healthy functioning during times of crisis. Even though a universal definition of resilience has been questioned (Pooley and Cohen, 2010; Southwick et al., 2014; Valiente et al., 2021), the literature agrees that two components must be present: First, the individual must encounter adversity or a high-risk situation and second, there must be a process of successful adaptation (e.g., Schilling, 2008). The result of this adaptation is often reflected in high well-being and is facilitated by a person’s resources, also called protective factors (Buchanan, 2014).

In the current study, we specifically focused on the role of adversities related to uncertainties regarding adolescents’ education and health as risk factors for adolescents’ mental health. Whereas adolescents were confronted with such objective stressors in the form of COVID-19-related school closures, we assumed that adolescents would react with subjective perceived stress, reflected in higher educational and health concerns. Regarding protective factors for adolescents’ mental health, we investigated the relative role of perceived social support by teachers, family, and friends. We first analyzed whether there were different groups of adolescents who could be described by different levels of risk (i.e., higher perceived stress reflected in higher educational and health concerns) and protective factors (i.e., perceived social support) associated with either lower or higher levels of mental health. These resilience profiles were examined within a person-centered approach. In addition, we used a variable-centered approach to investigate the relative associations of these risk and protective factors with adolescents’ mental health. Hereby, we first predicted adolescents’ mental health with the risk and protective variables; second, we investigated whether perceived social support by teachers, family, and friends buffered negative associations of educational and health concerns with adolescents’ mental health. The stress-buffering hypothesis proposes that perceived social support serves as a protective factor against negative effects of stress from negative life events on mental health (Cassel, 1976). High perceived social support may have been particularly important during school closures in early stages of the COVID-19 pandemic.

Lastly, we aimed to understand to what extent the exposure to the virus and related restrictions might have affected adolescents’ resilience with pandemic-related educational and health concerns by comparing adolescents from three different regions (i.e., German-speaking part of Switzerland, Italian-speaking part of Switzerland and Northern Italy).

### Adolescents’ Educational and Health Concerns During the Pandemic

The onset of the measures to contain the spread of the pandemic dramatically changed adolescents’ everyday lives and was associated with lower mental health (Jones et al., 2021; Stefaniak et al., 2021), such as increasing levels of depressive symptoms and loneliness (Grey et al., 2020). Particularly, young people seem to be at risk in such situations, as they react more strongly to surrounding stressors (El-Zoghby et al., 2020; Stefaniak et al., 2021) and are more vulnerable to traumatic and stressful events (Zhang et al., 2014). Stress may be either characterized as an external event, typically measured with negative life events believed to be stressful, and hence objective (Holmes and Rahe, 1967; Derogatis and Coons, 1993; Christensen et al., 2019) or it may be described as an individual perception central to the impact of a given stressor and hence subjective (Lazarus and Folkman, 1984; Christensen et al., 2019). The current study focused on subjective perceptions of stress, reflected in adolescents’ educational and health concerns, with higher concerns reflecting higher levels of perceived stress. We expected that these were two particularly salient concerns during early phases of the pandemic, when objective stressors such as lockdowns and school closures changed adolescents’ routines (de Miranda et al., 2020; Lee, 2020), and when relatively little was known about the new virus, potentially exacerbating fear of being infected (Fegert et al., 2020) and general concerns about the pandemic (Ellis et al., 2020).

#### Educational Concerns Associated With Pandemic-Related School Closures

Studies conducted prior to the pandemic suggest that adolescents experience stress due to increasing educational demands and concerns for their educational performance (Huan et al., 2008; Rickwood et al., 2016; Pascoe et al., 2020). Such educational concerns are associated with several undesirable outcomes. Among those are negative affect (Arsenio and Loria, 2014), negative general mood (Arsenio and Loria, 2014), lower self-esteem, higher anxiety and depression (Nguyen et al., 2019), and decreasing educational engagement (Reschly et al., 2008). Adolescents’ educational concerns have been investigated as symptoms of school burnout, which is defined by cynicism, exhaustion at school, and a sense of inadequacy at school (Bask and Salmela-Aro, 2013). Recent work shows that school burnout is associated with lower mental health (Nazar et al., 2020; Özhan and Yüksel, 2021) and lower educational achievement (Madigan and Curran, 2021).

Educational concerns may have been particularly salient during school closures associated with the COVID-19 pandemic. First, adolescents were deprived of routine habits associated with going to school, such as clear daily structures and regular interactions with peers and teachers and faced uncertainty and about future educational achievements (de Miranda et al., 2020; Golberstein et al., 2020; Hoffman and Miller, 2020; Wang et al., 2020; van Loon et al., 2021). Moreover, recent evidence pointed to increased worries about being behind and getting delayed in school (van Loon et al., 2021). Such educational concerns can increase loneliness, particularly when coupled with reduced social interactions (Loades et al., 2020; Wang et al., 2020). In addition, schools converted classroom teaching to distance learning, creating new challenges for both, teachers and students (Bond, 2020). The new educational setting required, for example, technology knowledge on the part of the teacher and technology acceptance on the part of the student (Bond, 2020), whereas such additional demands on new skills may have been overwhelming for certain students.

Taken together, school closures during the pandemic confronted students with new challenges. For certain adolescents, these sudden changes in the educational setting and educational demands might have exceeded their resources (Wang et al., 2020), increasing their educational concerns. Based on previous work (Wang et al., 2020; van Loon et al., 2021), we thus assumed that heightened educational concerns reflected a risk factor for adolescents’ mental health.

In addition to educational concerns, adolescents encountered health concerns. In March 2020, with the number of cases dramatically increasing, the development of the pandemic situation was still unknown. How infectious the virus was and how long the measures to contain the virus would continue were uncertain, potentially engendering pandemic-related health concerns.

#### Health Concerns Associated With the COVID-19 Pandemic

A recent systematic review pointed to a decline of adolescents’ mental health because of COVID-19-related health concerns (Meherali et al., 2021). For example, among Canadian adolescents, almost every other adolescent was “very concerned” about the pandemic, expressing high degrees of loneliness and depression (Ellis et al., 2020). Moreover, intolerance of uncertainty during the COVID-19 situation was directly and indirectly associated with negative emotions and higher risk perception among Chinese adolescents (Li et al., 2021). Relatedly, evidence suggests that the pandemic can increase the risk of post-traumatic stress symptoms (de Miranda et al., 2020; Guessoum et al., 2020; Liang et al., 2020) as well as anxiety and depressive symptoms (Duan et al., 2020; Fegert et al., 2020; Meherali et al., 2021). A German study comparing data before and during the pandemic, showed that the quality of life has decreased for adolescents, with the pandemic leading to higher levels of fear and mental disorders and lower mental health (Ravens-Sieberer et al., 2021). During the COVID-19 situation, adolescents might have also displayed other behavioral problems such as concentration problems, irritability, and reduced physical activity (Jiao et al., 2020), both potentially being related to adolescents’ health concerns during the lockdown. Based on this prior work, we assumed that health concerns would negatively relate to adolescent’s mental health.

While uncertainties related to school closures and health concerns were conceptualized as risk factors for adolescents’ mental health, social support by teachers, family, and friends was assumed to be an important protective factor for their mental health.

### Perceived Social Support

The current study focused on the role of perceived social support, consisting of an individual’s perception of how much support they feel they receive (Eagle et al., 2019; Qi et al., 2020). During adolescence, social support assumes a significant role for coping with developmental tasks associated with physical, emotional, and social changes (Pinkerton and Dolan, 2007). Evidence confirms that social support has a positive effect on social relationships and promotes feelings of being safe and cared for (Andrews et al., 2002). Furthermore, it has been found to alleviate anxiety, depression, and loneliness, with more perceived social support leading to better mental health (Grey et al., 2020). Evidence documents that high levels of perceived social support promote mental health at all points in life (Barker, 2007; Pinkerton and Dolan, 2007; Jakobsen et al., 2021). Thereby, the literature proposes that the more social support - received or perceived - a person has, the more they feel in control and the better they are able to cope with difficult situations, in particular (Szkody et al., 2020).

Based on these assumptions and relying on pre-pandemic and general literature about social support as well as based on recent literature examining social support during the pandemic (e.g., Ellis et al., 2020; Szkody et al., 2020; Campione-Barr et al., 2021; Bernasco et al., 2021; Ye et al., 2021), we expected that adolescents who perceived higher levels of social support during COVID-19 related school closures would report better mental health. Moreover, we investigated whether perceived social support buffered negative associations between educational and health concerns and mental health.

Social support has been found to be particularly helpful during stressful times, serving as a buffer by reducing negative effects of those events (Armstrong et al., 2005). Accordingly, the stress-buffering hypothesis proposes that perceived social support serves as a protective factor against negative effects of stress from negative life events on mental health (Cassel, 1976). Regarding the COVID-19 pandemic, recent literature found support for this hypothesis, whereby perceived social support (i.e., from friends, family, and someone close to the participant) buffered the negative association between worries about COVID-19 and psychological health (Szkody et al., 2020).

Importantly, previous studies show that different sources of social support may differentially relate to adolescents ‘mental health. For example, Colarossi and Eccles (2003) showed on the one hand that self-esteem was significantly predicted by adolescents’ perceived friend and teacher support but not by support of their mothers. On the other hand, perceived support from mothers had the largest effect for adolescents’ level of depression. The authors argue that parental support may have cumulative effects over time on depression because of long-standing and relatively stable parent-child relationships. Extending this prior work, an important aim of this study was also to assess the relative role of different sources of social support for adolescents’ mental health during the pandemic. During school closures, the intensity and nature of social interactions has changed considerably for many adolescents. Home-schooling and social distancing implied that adolescents spent a lot of time at home and less time with their friends and teachers at school. Under these uncommon circumstances, family support may have played a more decisive role for adolescents’ mental health during school closures than perceived teacher and friend support.

#### Teacher Support

With a large portion of daily life spent at school, teachers play an important role in adolescents’ life. The literature conceptualizes teacher support as teachers being sensitive to their students’ needs (Hamre and Pianta, 2007). Especially when paired with consistency (i.e., stable and predictable support), teacher support helps making students feel secure and giving them the confidence to be more active in school, socially as well as academically (Curby et al., 2013). Teacher support thus seems to have the potential of promoting adolescents’ social and educational development. Research has shown that teacher support is indeed linked with the use of self-regulatory strategies (Wang and Holcombe, 2010) and more prosocial behavior (Farmer et al., 2011). Students perceive teachers to be particularly supportive when they feel an emotional bond and when teachers support a fair environment that recognizes and praises educational success (Suldo et al., 2009). The conceptualization of teacher support usually distinguishes two components: emotional support and instrumental support. The former entails that teachers show to students that they care about them and the latter consists of making sure students have everything they need to learn (Suldo et al., 2009).

The COVID-19 pandemic has led to substantial changes and challenges in how teachers provide their students with much-needed social interactions and emotional bonds during school closures (Ye et al., 2021). Teachers and students reported being overwhelmed with new teaching approaches and technologies (Beteille et al., 2020), likely to engender uncertainty about student-teacher relationships on both sides. Teacher support may not only have been an important source for students in dealing with the acquisition of new skills for remote learning, but also a source for dealing with uncertainty about the academic situation. Accordingly, a recent study by Moser et al. (2021) demonstrated that teachers expressed concerns about students’ achievements. Moreover, students felt a lack of consultation (Korzycka et al., 2021) and decreased support from teachers (Lessard and Puhl, 2021).

In contrast, high support from teachers has shown to be beneficial for adolescents’ engagement in remote learning during school closures (Bray et al., 2021). Similarly, a study from Garrote et al. (2021) showed that if teachers had high expectations on adolescents’ remote learning performance, students performed better in general and had a more positive perception of distance learning. Thus, high perceived teacher support may have been an important resource during school closures for adolescents’ mental health as well as serving as a buffer against subjective stressors. A recent study showed that a positive student-teacher relationship was associated with higher academic engagement as well as fewer mental health problems in times of online learning as schools were closed (Ye et al., 2021). In addition, these authors also showed that positive student-teacher relationships buffered negative effects of cyberbullying and difficulties with COVID 19-related online learning on mental health.

Based on these first insights, we assumed that teachers who provided high support to students during school closures promoted students’ well-being during the situation and helped to buffer potential negative associations for their mental health.

#### Family Support

In addition to teachers, family support is regarded as essential for adolescents’ psychological adjustment (Anderson et al., 2007) and mental health (Anthony and Stone, 2010). Parents are seen as the closest source of help for adolescents in terms of reducing and coping with stressors and thus promoting well-being (Guessoum et al., 2020). For example, good parental communication (i.e., positive communication and the willingness to seek parental advice) and family dinners have positive effects on adolescents’ psycho-social development, with adolescents displaying lower depression, more engagement in learning and less school problems, and more positive social behavior (Fulkerson et al., 2006). Therefore, more time spent with the family may help reduce the effect of stress on adolescents’ mental health.

These findings suggest that adolescents’ families may have been central sources of social support during the COVID-19 pandemic, particularly during school closures. Especially warm, supportive, and democratic parenting styles were found to have a positive effect on adolescents’ mental health, as opposed to a more authoritarian style (Ye et al., 2021). Regarding school closures, parents’ reactions to the pandemic have shown to have an effect on adolescents’ adjustment to online learning, with less parental stress correlating with a more positive experience of adolescents’ online learning during school closures (Garrote et al., 2021). Recent evidence with Dutch adolescents showed an increase in parental support with a simultaneous decrease of anxiety and depression during a 20-day period of at first online, and later on, mostly physical school days in times of the pandemic (Klootwijk et al., 2021). In addition, Ellis et al. (2020) documented that spending more time with the family (i.e., time spent with family activities during the past three weeks) was associated with higher mental health among Canadian adolescents.

In contrast, a recent study found that a sizeable proportion of adolescents (36% of the sample) spent less than 30 minutes per day with their family during the COVID-19 pandemic (Ellis et al., 2020). Other studies even reported an increase in domestic violence during the lock-down (Chandan et al., 2020; Imran et al., 2020; Lee, 2020) with an increase of conflict occurring between adolescents and their parents, leading to lower life satisfaction and, regarding conflicts with fathers in particular, more depressive symptoms (Magson et al., 2020). Such negative interactions at home are thought to negatively affect adolescents’ mental health.

Based on these previous insights, we assumed that high perceived family support played a central role for adolescents’ mental health during school closure. Moreover, based on the stress-buffering hypothesis, we assumed that perceived family support may have buffered negative consequences of adolescents’ educational and health concerns for their mental health. This assumption was also based on previous insights of the positive role of parental support for adolescents’ learning (Chohan and Khan, 2010; Shukla et al., 2015) and their important function of providing emotional security to their children (Fulkerson et al., 2006). In line with this, recent literature pointed out that high family support served as a buffer of loneliness as a reaction of how severe COVID-19 was perceived (Wang et al., 2021).

#### Friend Support

During adolescence, individuals spend less time at home and generally socialize more often with friends; thus, interactions with friends increase in importance for adolescents’ psycho-social development (Rubin et al., 2006; Wang et al., 2011). Friend support, as one of the sources of social support, describes a variety of connections (e.g., emotional support or help-seeking) built with friends that have an influence on a person’s functioning (Barker, 2007). Several studies documented the benefits of friend support by showing that it is positively associated with social competence, higher self-esteem, lower depression and lower stress levels (Wilburn and Smith, 2009), higher self-regulation (Patrick et al., 2007), and better psychosocial mental health (Vieno et al., 2007; Williams and Anthony, 2015; Traylor et al., 2016).

During school closures and social distancing rules in the COVID-19 pandemic, adolescents faced strong restrictions for personal meetings with their friends. Magson et al. (2020) found that not being able to see their friends as much was a great concern for many adolescents; feelings of social disconnection were associated with higher levels of symptoms of anxiety and depression and less life satisfaction (Magson et al., 2020). However, many adolescents met their friends online: Ellis et al. (2020) investigated how time spent with friends virtually was associated with adolescents’ mental health during the COVID-19 pandemic and found that more time spent with friends online was linked to lower loneliness on the one hand, but at the same time associated with greater depression. Similarly, Bernasco et al. (2021) investigated whether time spent with friends either online or offline moderated the association between pre-COVID friend support and stress related to COVID. However, they didn’t find such a moderating effect, whereas pre-COVID support from friends predicted having less COVID-related stress. In contrast, Campione-Barr et al. (2021), demonstrated that close relationships to best friends predicted adolescents’ ability to adjust to pandemic-related concerns (i.e., by showing lower levels of anxiety, depressive symptoms, problem behavior) while controlling for their pre-pandemic adjustment.

These findings highlight the importance of perceived friend support during the COVID-19 pandemic, suggesting that lower perceived friend support may be associated with lower mental health, either directly, or as buffer for negative experiences.

In the current study, we assumed that the family may have become relatively more important for adolescents’ mental health during the pandemic in comparison with friends and teachers. First insights regarding the relative role of different social support systems during the COVID-19 lockdown with an adult sample from Egypt show that the need of familial support and of caring for family members has increased during times of isolation, whereby almost twice as many participants reported increased support from family members as compared to increased support from friends (El-Zoghby et al., 2020). Similarly, Ellis et al. (2020) showed that spending more time with the family, actively as well as virtually, was associated with fewer depression, whereas findings regarding time spent with friends virtually were mixed, with adolescents reporting lower loneliness but greater depression instead. In addition, Campione-Barr et al. (2021) illustrated that when adolescents encountered high COVID-related stress, they experienced greater problem behavior even when perceiving high positivity in their friendships, while positive relationships with parents predicted adolescents’ adjustment (Campione-Barr et al., 2021).

### Regional Differences

The sample of the current study included three different regions (i.e., German-speaking part of Switzerland, Italian-speaking part of Switzerland, and Northern Italy) in order to investigate possible regional differences in the level of exposure to the COVID-19 virus and the measures taken to contain its spread. In the literature, heterogeneous findings regarding possible regional differences associated with the pandemic were found. For example, no regional differences were found in the stress level and mental health in two different studies on Italian adolescents (Commodari and La Rosa, 2020; Nocentini et al., 2021). In contrast, two recent studies suggested that living in a high-risk and strongly affected area may influence mental health, with the prevalence of post-traumatic stress symptoms being higher for people living in such areas (Liu et al., 2020), and lower mental health of students living in more strongly affected regions in China (Wu et al., 2021). Moreover, living in a strongly affected area or an urban area were two factors associated with higher anxiety level and depression symptoms in Chinese adolescents (Duan et al., 2020). We thus explored whether there were regional differences in adolescents’ pandemic-related educational and health concerns, which may have been more salient in regions with higher exposure to the virus and more restrictive measures.

### The Current Study

Based on a resilience framework, the current work aimed to identify different profiles of adolescents’ risk and protective factors being associated with mental health during the beginning of the COVID-19 pandemic, when schools were closed, strong restrictions on social interactions were imposed, and high levels of uncertainty pertained to negative consequences and the spread of the virus. In order to better understand the relative role of educational concerns and health concerns as risk factors and the relative role of perceived social support by teachers, family, and friends as protective factors for adolescents’ mental health, we employed a person-centered approach to identify different groups of adolescents with either higher or lower resilience profiles.

Moreover, we investigated whether adolescents living in regions where different measures were imposed to contain the spread of the virus differed in their likelihood to belong to one of the resilience profiles, while accounting for socio-demographic differences. Lastly, in addition to the exploratory approach of identifying resilience profiles, we also used a variable-centered approach to examine the relative role of risk and protective factors for adolescents’ mental health (i.e., current well-being and depressive mood) as well as a potential moderating, stress-buffering role of perceived social support for adolescents against subjective stress, reflected in their educational and health concerns.

Regarding mental health indicators, we included adolescents’ current well-being during the lockdown as well as their depressive symptoms, with one aspect reflecting a more situational measure, capturing well-being during the pandemic, and depressive mood reflecting a general condition over a longer period of time. Adolescents with higher depressive mood may have had lower resources to deal with the challenges related to the pandemic. Accordingly, recent evidence identified pre-pandemic psychiatric disorders (Becker and Gregory, 2020; Guessoum et al., 2020; van Loon et al., 2021), pre-pandemic stress (van Loon et al., 2021), maladaptive coping skills (Guessoum et al., 2020; van Loon et al., 2021) or sleep problems (Becker and Gregory, 2020) as additional risk factors for low mental health during the COVID-19 pandemic.

#### Hypotheses Regarding Adolescents’ Resilience Profiles

We assumed that the relative level of risk and protective factors as well as current well-being and depressive mood (i.e., mental health indicators) would generate qualitatively and quantitatively differences between different groups of adolescents, crystallizing in different resilience profiles. We did not expect a specific number of profiles. Regarding quantitative differences, we assumed that educational and health concerns were at lower levels in profiles reflecting high resilience (e.g., Wang et al., 2020; Ellis et al., 2020). Moreover, we assumed that high resilience profiles would be characterized by high levels of perceived social support (e.g., Chu et al., 2010; Fegert et al., 2020; Szkody et al., 2020; Ye et al., 2021) as well as high current well-being and lower levels of depressive mood (e.g., Loades et al., 2020). For profiles with low resilience, we expected high levels in both risk factors (i.e., educational and health concerns), low levels of perceived social support and low current well-being as well as higher depressive mood.

In addition to these quantitative differences, we also anticipated qualitative differences between the profiles. Here, we first explored whether there were qualitative differences between educational and health concerns within the profiles. Moreover, we expected the three components of perceived social support (i.e., perceived teacher, family and friend support) to differ in their relative importance in times of the lockdown (e.g., Ellis et al., 2020; Prime et al., 2020; Campione-Barr et al., 2021). Based on the hours spent at home during the lockdown, we expected perceived family support to vary the most between the resilience profiles among all three sources of social support.

#### Hypotheses Regarding Regional Differences

In addition to investigating adolescents’ resilience profiles, an important aim was to explore whether different levels of exposure to the virus and according measures taken predicted adolescents’ resilience profiles. Therefore, adolescents from three regions differentially affected by COVID-19 at the onset of the pandemic were compared, namely, from the German-speaking part of Switzerland, the Italian-speaking part of Switzerland, and Northern Italy (i.e., Lombardy).

On March 16, 2021, with the quick spreading of the COVID-19 virus, Switzerland decided to close schools, shops, and restaurants, resulting in a significant decrease in daily incidence. At the End of March, as the situation became more critical, each Swiss region could individually decide whether to take extra measures. Compared to the German-speaking part, the Italian-speaking part of Switzerland adopted more severe restrictions, as COVID-19 hit this region more strongly. This region decided to prolong the closure of trading and production activities. The Federal Council decided to gradually reduce these measures by the end of April, with hospitals allowing non-urgent surgery to take place and with schools reopening for classroom teaching on May 11, 2020 (Bundesamt für Gesundheit, 2020; Forster, 2020).

Meanwhile, Northern Italy was the most affected area, especially at the onset of the pandemic. Seven Italian regions, including Lombardy, have decided to close their schools as of 24 February, while the Italian government has decided to close schools throughout Italy as of 5 Marchuntil September, offering home-schooling whenever possible and isolating some regions particularly at risk (Caffo et al., 2020; Ministero della Salute, 2021). While in Switzerland it was possible to leave the house and meet friends (up to 5 people) with necessary precautions such as the social distance, the restrictions in Italy were harder, and it was not possible to move freely between the regions or leave the house, if not for some necessity, such as grocery shopping. Comparing the three regions at the time of the survey (around mid-April), schools were closed in all three regions, with the Italian-speaking part of Switzerland and Italy having more cases than the German-speaking part of Switzerland. In addition, Italy had stricter restrictions concerning the possibility of leaving the house, not allowing inhabitants to go out beside for some exceptions (e.g., groceries), while Switzerland allowed gatherings of up to 5 people.

Considering the case numbers and measures taken, the exposure to the virus is arguably depended on the country and, more specifically, on the region. Therefore, we explored whether regions with higher exposure (i.e., Italian-speaking part of Switzerland and Northern Italy) were more likely to belong to profiles with higher risk factors, lower resources and lower mental health, as compared to areas with lower exposure (i.e., German-speaking part of Switzerland).

In these analyses, we controlled for grade, gender, socio-economic status [SES], and migration background, as previous evidence pointed to differences in mental health depending on these variables (de Miranda et al., 2020; Masonbrink and Hurley, 2020; Zhou et al., 2020; Nocentini et al., 2021; Ravens-Sieberer et al., 2021).

#### Hypotheses Regarding the Role of Risk and Protective Factors for Adolescents’ Mental Health

We analyzed within a variable-centered approach whether educational and health concerns and perceived social support predicted adolescents’ mental health, reflected in their current well-being and depressive mood. Similar to the hypotheses regarding the resilience profiles, we assumed negative associations between educational and health concerns with mental health and positive associations of all sources of perceived social support with adolescents’ mental health (e.g., Grey et al., 2020; Bernasco et al., 2021; Bray et al., 2021; Jakobsen et al., 2021; van Loon et al., 2021; Ye et al., 2021). Regarding the relative role of educational and health concerns, we investigated whether these predictors differed in their association with both aspects of mental health. Regarding the relative role of social support, we specifically hypothesized that perceived family support would be more predictive of adolescents’ mental health rather than perceived teacher and friend support (e.g., Ellis et al., 2020; Campione-Barr et al., 2021; Wang et al., 2021). Lastly, based on the stress-buffering hypothesis (Cassel, 1976; Szkody et al., 2020), we explored moderating relations of all three sources of social support between educational and health concerns and mental health.

## Methods

### Participants and Design

Cross-sectional data was collected in two regions of Switzerland and in Northern Italy during three weeks in mid-April of 2020. Hence the data was collected in the beginning of the COVID-19 pandemic, when schools had been closed in all three regions as one of the measures to contain the spread of the virus.

This study was conducted in accordance with ethical standards of the APA and was approved by the Ethics Committee of the[blinded]. The analyses are based on the data from 1,562 students with an average age of 16.18 years (*SD* = 1.48, range = 14-20 years). Seventy-two percent of the participants specifying their gender reported identifying as female (28% as male). At the time of the survey, most of the participants were in grade nine (21%), ten (20%), or eleven (19%) and mostly attending high school (58%) or secondary school (26%). The rest of the sample were either in an apprenticeship (12%), in middle school (3%) or in a higher technical school (1%).

With 80%, the largest proportion of participants lived in Switzerland (49% in the Italian- and 31% in the German-speaking part of Switzerland) and 20% of the participants lived in Northern Italy. Furthermore, 27% of the participants indicated another nationality than or an additional nationality to the Swiss one (i.e., for the Swiss participants) or the Italian one (i.e., for the Italian participants). To assess participants’ socio-economic status (SES), their housing situation was assessed. Seventy-five percent of the students stated that they lived in an owned house or apartment and 25% stated that they lived in rented accommodation. For details about the demographic information for the total sample as well as for the specific regions in which the survey was conducted, see Supplementary Table 1 in the Supplementary Material.

The data was collected via an online survey (approx. 20 min). Schools received a link to the questionnaire and forwarded it to their students if they agreed to participate in the study. Before taking the survey, the participants were informed that their participation was voluntary and that their data was anonymous. After completing the survey, participants received gift certificates.

### Measures

The item correlations and respective reliability measures of the scales can be found in Table 1. Supplementary Table 2 in the Supplementary Material contains a complete list of all items. All items were rated on a six-point-scale (0 = completely disagree, 5 = completely agree).

**TABLE 1:** Descriptive statistics of the components of the resilience profiles and the control variables (*N* = 1562).

	*M (SD)*	(1)	(2)	(3)	(4)	(5)	(6)	(7)	(8)	(9)	(10)	(11)
(1) Educational concerns	2.35 (1.15)	0.76										
(2) Health concerns	3.63 (1.01)	0.10^***^	0.84									
(3) Perceived teacher support	3.06 (1.03)	–0.42^***^	0.05^†^	0.82								
(4) Perceived family support	3.62 (1.10)	–0.32^***^	0.15^***^	0.37^***^	0.86							
(5) Perceived friend support	3.70 (0.91)	–0.15^***^	0.10^***^	0.21^***^	0.21^***^	0.83						
(6) Current well-being	3.13 (0.82)	–0.51^***^	–0.07^**^	0.33^***^	0.40^***^	0.18^***^	0.74					
(7) Depressive mood*	2.58 (1.23)	–0.47^***^	–0.15^***^	0.23^***^	0.35^***^	0.06*	0.56^***^	0.78				
(8) Grade	10.19 (1.52)	0.23^***^	0.03	–0.29^***^	–0.15^***^	−0.05^†^	–0.23^***^	–0.18^***^	−			
(9) Gender	0.72 (0.45)	0.10^***^	0.22^***^	–0.11^***^	−0.05^†^	0.11^***^	–0.15^***^	–0.21^***^	0.16^***^	−		
(10) SES	0.75 (0.43)	0.00	0.04	–0.04	0.00	–0.01	0.04	–0.04	0.13^***^	0.04	−	
(11) Migration background	0.27 (0.44)	–0.04	–0.01	0.03	–0.02	−0.05^†^	0.00	0.03	–0.14^***^	–0.08^**^	–0.34^***^	−

Means (M) and standard deviations (SD) are shown in the first column. Range of the scales of the 7 components: 0 (completely disagree) to 5 (completely agree); gender: 1 = female, 0 = male; SES: 1 = own house, 0 = rented house; Migration background: 1 = migration background, 0 = no migration background. The reliability of the scales is reported in the diagonal. * Depressive mood was recoded, with higher levels reflecting lower depressive mood.

^†^p < 0.10, *p < 0.05, ^**^p < 0.01, ^***^p < 0.001, two-tailed.

#### Educational Concerns

Educational concerns were measured with four items capturing adolescents’ academic fears and worries from the school burnout questionnaire that focus on concerns regarding school and education (e.g., “I feel overwhelmed with school.”, “I am afraid that I will have to repeat the school year”; adapted from Satow and Mittag, 1999; Salmela-Aro et al., 2009).

#### Health Concerns

Health concerns were operationalized with five items regarding concerns for the health of oneself or others related to COVID-19, adapted from measures developed in studies on previous pandemics (e.g., “I am concerned that my friends or family may become seriously ill because of Corona.”, “I am worried that Corona continues to spread”; adapted from Han et al., 2014 and Wong and Tang, 2005). For the profiles, all scales were recoded, with higher scores representing lower levels of educational concerns and lower levels of health concerns, respectively.

#### Perceived Social Support by Teachers, Family, and Friends

Perceived social support was assessed using three previously validated scales, thereby examining three different components of emotional support for students: teachers, family, and friends. One of the three items regarding perceived teacher support was e.g., “My teachers always help me when I get stuck.”, “My teachers notice when I have a problem.” (see Ryzin et al., 2009; Gasser et al., 2018). The measures of perceived family support and perceived friend support also consisted of three items each. These two scales only differed with regard to the source of support (e.g., “I can always rely on my family.” resp. “I can always rely on my friends.”, “I can tell my family everything” resp. “I can tell my friends everything”; adapted from Procidano and Heller, 1983; Satow, 2012).

#### Mental Health

Two components were used to assess participants’ mental health. The first scale was a measure of current well-being, focusing on the previous week. In particular, adolescents were asked how they were doing in quarantine and how their last week was. It consisted of five items (e.g., “I felt happy.”, “I was full of energy.”; Ravens-Sieberer et al., 2010). The second scale included three items regarding depressive mood reflecting a rather stable measure of mental health. In particular, adolescents reported whether they tended to be in certain states over a longer period of time (e.g., “I often feel sad or unhappy.”, “I often feel lonely”; Wentzel et al., 1990). This scale was recoded to have higher scores representing higher mental health in both scales.

#### Regions and Socio-Demographic Variables

*Regions.* Regions were assessed as a factor variable, including two areas from Switzerland (i.e., German- and Italian-speaking parts), and one region in Northern Italy (i.e., Lombardy).

*Socio-Demographic Variables (Control Variables).* Adolescents reported their grade attending at the time of the survey. Given that data on age was limited to year of birth and that our central questions focused on educational concerns, grade was considered a more accurate control variable than age. Gender was coded as either female or male (1 = female, 0 = male). SES was conceptualized as a dummy variable reporting the housing situation of adolescents (1 = owned house, 0 = rented house). Migration background was operationalized as a dummy variable, whereby reporting another nationality than or indicating an additional nationality than the resident country were used as indicators of migration background (i.e., Swiss for the samples from the German- and Italian-speaking parts of Switzerland and Italian for the sample from Northern Italy).

### Data Analytic Strategy

The current study was based on a combination of a person-centered approach, including latent profile analysis (LPA), and a variable-centered approach, including a path analysis (PA) and multinomial logit modeling (MLM).

The goal of the LPA was to find out whether different latent profiles emerged for different groups of adolescents. Such profiles reveal quantitative and qualitative differences in the specific variables between subpopulations of adolescents. To identify profiles, we used in total seven components (i.e., educational and health concerns, three different components of perceived social support and two different components of mental health). Thus, with regards to our research questions, the analyses helped to investigate whether the relations among the three main constructs were differently associated for certain groups of adolescents.

The LPA was executed with the *MPlusAutomation* package (Hallquist and Wiley, 2018), using R-Studio via *Mplus* (Muthén and Muthén, 2018). To select the optimal number of classes, we started with a solution of one class and subsequently increased the number of latent profiles, whereby we compared the Bayesian information criterion (BIC, with lower values indicating a better fit), the entropy value (i.e., the confidence with which individuals can be classified into a specific profile, ranging from 0-1, recommended > 0.8) and the Lo-Mendell-Rubin (LMR) test. The LMR test tests the hypothesis that K-classes are optimal compared to K-1 classes (Lo et al., 2001). In addition, we considered the interpretability of the different profiles, particularly how well the profiles differentiated between groups and whether they differed quantitatively (i.e., in their level) or also qualitatively (i.e., in their pattern). To facilitate model convergence, variances across profiles were freely estimated and covariance constrained to be unrelated to one another (i.e., constrained to 0). Moreover, in order to ease the interpretation of the profiles, the scales were mean-centered. We accounted for missing data by using full maximum-likelihood estimation (FIML) in *Mplus 8.6* (Muthén and Muthén, 2018).

Next, in order to understand whether educational and health concerns on the one hand and perceived social support on the other hand were associated with adolescents’ mental health at a general level, we investigated the associations between the key variables within a path analysis (PA). Within this approach, we specified contrast between the relative predictive value (i.e., the regression parameters) of educational and health concerns, as well as between different sources of social support as additional parameters with the “model constraints” in MPLUS. Thus, we were able to answer the question whether educational and health concerns respective social support by teachers, family, and peers, differed in their relative association with mental health. Lastly, we investigated whether perceived social support moderated the association between educational and health concerns and mental health in order to test whether perceived social support buffered negative consequences of stress during the school closures associated with the COVID-19 pandemic.

Lastly, as we were interested in regional differences, we tested whether the profiles could be generalized for the different regions (as recommended by Morin et al., 2016). Thus, before we investigated whether adolescents from different regions had different probabilities of belonging to the profiles identified for the complete sample, we first investigated, whether we could replicate the profiles within the regional subsamples. As we were able to replicate the profiles for the three subsamples (see Supplementary Material), we computed a MLM, whereby we entered the region (i.e., German-speaking part of Switzerland, Italian-speaking part of Switzerland and Northern Italy) as predictor for profile membership. As we had a special interest in regional differences, we controlled for individual variables (i.e., grade, gender, SES and migration background).

## Results

Descriptive results are displayed in Table 1. The descriptive data shows that the mean of educational concerns, *M* = 2.35, *SD* = 1.15, was somewhat lower than the mean of health concerns, *M* = 3.63, *SD* = 1.01. Regarding social support the mean of perceived friend support, *M* = 3.70, *SD* = 0.91, was slightly higher than the mean of perceived family support, *M* = 3.62, *SD* = 1.10, whereas adolescents had relatively lower perceptions of teacher support, *M* = 3.06, *SD* = 1.03. Interestingly, health concerns were significantly positively correlated with perceived teacher support, *r* = 0.05, *p* < 0.1, with perceived family support, *r* = 0.15, *p* < 0.001, and with perceived friend support, *r* = 0.10, *p* < 0.001, which implies reporting higher health concerns was associated with perceiving higher support from teachers, families and friends. In contrast, educational concerns were as one would expect negatively correlated with all three sources of perceived social support (teachers, *r* = -0.42, *p* < 0.001, families, *r* = -0.32, *p* < 0.001, friends, *r* = -0.15, *p* < 0.001).

### Latent Profile Analysis

**FIGURE 1:**
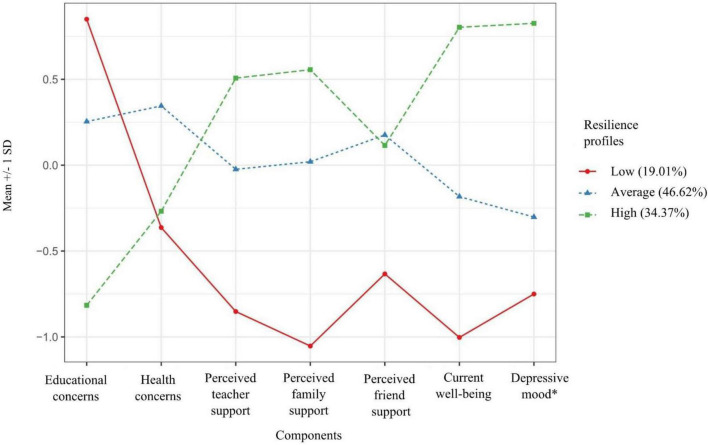
Latent resilience profiles. Scales marked with an asterisk * were recoded so that higher values of depressive mood reflect lower depressive levels. All components were mean-centered.

When deciding about the number of profiles in a stepwise procedure, fit information revealed that a solution with two profiles fit the data well. In addition, a three-profile solution lead to a considerable improvement of the log likelihood and BIC value while the LMR test was significant (see Table 2). A four-profile model did not lead to a considerable higher improvement and also had a non-significant LMR test. When examining the profile plots (showing the mean-centered values for each variable within each profile) for the model with two respective three profiles, the two-profile model consisted of two predominantly quantitatively different profiles with low, respective high resilience (see Supplementary Figure 1) while the three-profile model also included a group with average resilience. In the two-profile solution 50% were identified being in the low resilience profile and 50% of the sample belonged to the high resilience profile. The three-profile solution (minimal class probability = 0.89, maximum class probability = 0.91) included three latent resilience profiles (see Figure 1): A low (19% of the sample), an average (47% of the sample) and a high (34% of the sample) resilience profile. Based on the fit values and the information from the profile plots, we decided that the three-profile model did the best job in explaining the heterogeneity in adolescents’ resilience. Moreover, as one of our aims was to investigate regional differences, a three-profile solution was generalizable across subsamples. We estimated and plotted the profiles for the German- and the Italian-speaking part of Switzerland as well as Northern Italy separately. Results revealed that the three-profile solution showed similar patterns within each region as it did in the entire sample (see Supplementary Figures 2, 3, and 4 in the Supplementary Material).

**TABLE 2:** Fit information of the latent profile analysis.

*No of classes*	*Log Likelihood*	*BIC*	*Entropy*	*LMR p-value*
**LPA**				
1	−13297.63	26698.21	1	
2	−12637.35	25487.95	0.67	0.000
3	−12419.41	25162.38	0.64	0.012
4	−12284.77	25003.41	0.67	0.311

*BIC = Bayesian information criterion; LMR = Lo-Mendell-Rubin Test; LPA = latent profile analysis.*

The findings of the three-profile solution were in line with our hypothesis, assuming that there would be theoretically cohesive latent resilience profiles, with the three dimensions concerns, perceived social support, and mental health being correlated for each group at either a low, average or high level. Supporting our hypotheses, adolescents belonging to the low resilience profile were characterized by high levels of educational concerns, low levels of perceived social support (i.e., low perceived teacher support, low perceived family support, and low to average perceived friend support) and low mental health (i.e., low current well-being and high levels of depressive mood). Belonging to the average resilience profile implied perceiving average levels of all seven components. In contrast, adolescents who were in the high resilience profile perceived low levels of educational concerns, high levels of perceived social support (high perceived teacher and family support, average perceived friends support) and high levels of mental health.

Regarding perceived social support, the means of perceived family and teacher support were about one *SD* below the mean for the low resilience profile and about 0.5 *SD* above the mean for the high resilience profile. This suggests that those sources of support were perceived as particularly low among adolescents in the low resilience profile. Moreover, with regards to relative differences in perceived support between the profiles, perceived teacher and family support varied more between the resilience profiles than perceived friend support. These qualitative differences suggest that support by families and teachers may have been relatively strong determinants of adolescents’ resilience profiles. While we expected this pattern for the family, we did not hypothesize that perceived teacher support and perceived friend support differed qualitatively from one another. In contrast, perceived friend support was relatively low in the low resilience profile compared to both the average and the high profile but did not differ between these two profiles. Hence, low perceived friend support correlated with lower mental health among adolescents in the low resilience profile.

Regarding health concerns, not all profiles differed in the expected way: for the low resilience profile, the level of health concerns was at a similar level as the high resilience profile. Thus, health concerns may have been less relevant than educational concerns for adolescents in the low resilience profile.

### Path Analysis

With the path analysis, we aimed to shed light on how the key variables related to each other. While the latent profile analysis identified different associations between these variables for different groups of adolescents, the results of the path model applied to the whole sample. In other words, while the latent profile analysis identified different resilience profiles based on different combinations of risk and protective factors, the current analysis investigated the general associations between the two risk (i.e., educational and health concerns) and three protective factors (i.e., perceived teacher, family, and friend support) with adolescents’ mental health.

**FIGURE 2:**
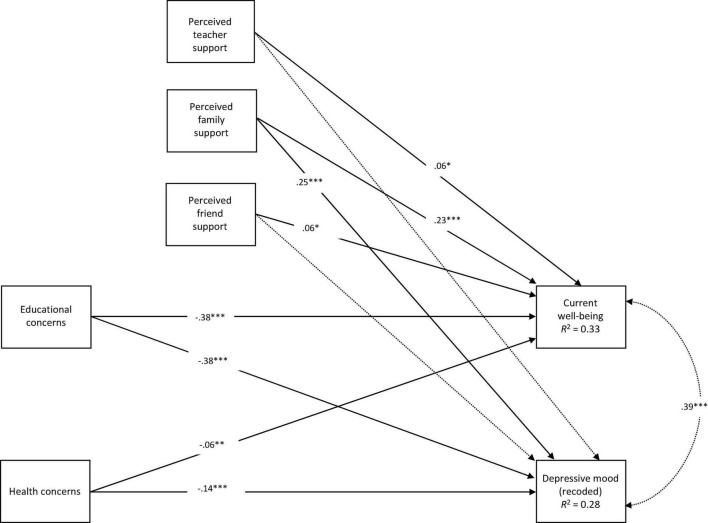
Path analysis predicting adolescents’ current well-being and depressive mood with the two risk (i.e., educational and health concerns) and three protective factors (i.e., perceived social support by teachers, family, and friends). Depressive mood was recoded, with higher values reflecting lower depressive mood. Standardized estimates are reported on the straight and curved arrows. The dashed arrows represent non-significant paths. *p < 0.05, ^**^p < 0.01, ^***^p < 0.001, two-tailed.

In a first step, we entered the risk and protective factors as predictors of both mental health, indicators, while accounting for the correlation of the two mental health measures. We included additional contrasts between educational and health concerns as well as between all three sources of social support as additional model constraints in order to test whether the effects differed in magnitude. The results (see Figure 2) showed that educational and health concerns were both associated with lower mental health. Moreover, significant contrasts between the regression parameters revealed that health concerns were less predictive for both mental health predictors than educational concerns (current well-being: Δ*b* = -0.45, *SE* = 0.03, *p* < 0.001; depressive mood: Δ*b* = -0.52, *SE* = 0.03, *p* < 0.001). Perceived family support was significantly and positively related to current well-being and depressive mood (which was recoded, with higher value displaying lower depressive mood), while teacher and friend support were only significantly associated with current well-being, but not depressive mood (see Figure 2). In line with our hypothesis, perceived support by family was more predictive for both aspects of mental health than perceived teacher (current well-being: Δ*b* = 0.19, *SE* = 0.04, *p* < 0.001; depressive mood: Δ*b* = 0.26, *SE* = 0.04, *p* < 0.001) and perceived friend support (current well-being: Δ*b* = 0.19, *SE* = 0.04, *p* < 0.001; depressive mood: Δ*b* = 0.28, *SE* = 0.04, *p* < 0.001). There was no significant difference between the regression parameters of perceived support by teachers and by friends and the two aspects of mental health. Importantly, the results did not differ, when including control variables.

Next, in order to explore whether social support served as a stress-buffer for mental health, we included the interaction terms of educational and health concerns with all three sources of social support to the model. Thereby, the only significant interaction parameter related to the interaction of educational concerns and perceived teacher support on depressive mood (β = 0.07, *SE* = 0.03, *p* = 0.02). However, when adjusting for multiple comparisons, this effect was non-significant.

Lastly, we explored whether there were regional differences between the hypothesized associations. Thereby, a multi-group model with the region as grouping variable in which all predictive regression parameters were constrained to be equal did not fit the data better than a model in which these parameters were freely estimated Δχ^2^ (22) = 26.45. Hence, the associations between the main variables did not differ between the three regions.

### Multinomial Logit Models

Our third aim of this study was to find out whether regional differences would predict adolescents’ likelihood of being classified in one of the three identified profiles.

To investigate this question, we performed multinomial logit models. As the high resilience profile was chosen as a reference category, these models compared the likelihood of belonging to the low respective the average resilience profiles relative to the high resilience profile. Thereby, we first included individual control variables. The results revealed that adolescents from higher grades, β = 0.48, *SE* = 0.06, *p* < 0.001, females, β = 0.53, *SE* = 0.20, *p* < 0.01, students with lower SES, β = -0.45, *SE* = 0.22, *p* < 0.05 or having a migration background, β = 0.39, *SE* = 0.21, *p* < 0.1, were more likely to belong to the low resilience profile as compared to the high resilience profile relative to adolescents from lower grades, males, students with higher SES and having no migration background (see Table 3). This was also the case for the average resilience profile, except that the coefficients on SES and migration background were not significant.

**TABLE 3:** Results of the multinomial logit model on resilience profile classification.

	Step 1				Step 2			
	Low resilience		Average resilience		Low resilience		Average resilience	
	β *(SE)*	*exp(*β)	β *(SE)*	*exp(*β)	β *(SE)*	*exp(*β)	β *(SE)*	*exp(*β)
Grade	0.48(0.06)***	1.62^***^	0.32(0.05)***	1.37^***^	0.38(0.06)***	1.46^***^	0.22(0.05)***	1.25^***^
Gender	0.53(0.20)**	1.69^**^	0.79(0.15)***	2.20^***^	0.44(0.21)*	1.55*	0.69(0.16)***	1.99^***^
SES	−0.45(0.22)*	0.64*	−0.19(0.17)	0.83	−0.63(0.22)**	0.53^**^	−35 (0.17)*	0.70*
Migration background	0.39(0.21)†	1.47^†^	−0.03(0.16)	0.97	0.51(0.21)*	1.66*	0.09 (0.17)	1.10
*Regional differences*								
Italian-speaking part of Switzerland					0.96(0.22)***	2.61^***^	0.71(0.15)***	2.03^***^
Northern Italy					2.05(0.32)***	7.81^***^	1.83(0.25)***	6.22^***^
AIC	2280.12				2209.22			
BIC	2330.71				2280.04			

*Reference category for the dependent variable = high resilience profile; reference category for regional differences = German-speaking part of Switzerland; gender: 1 = female, 0 = male; SES: 1 = own house, 0 = rented house; migration background: 1 = migration background, 0 = no migration background. The coefficients β are the logarithms of the ratio of the probability of choosing one outcome category over the probability of choosing the baseline category. Exp(β) is the exponentiation of the coefficients β, which can be interpreted as the relative risk or likelihood of belonging to the low or medium resilience profile relative to the high resilience profile when increasing the predictor variables by one unit or switching the category of the predictor.*

^†^p < 0.10, *p < 0.05, ^**^p < 0.01, ^***^p < 0.001, two-tailed.

In a second step, regions were added to the model, whereby significant regional differences emerged: Adolescents living in the Italian-speaking part of Switzerland, β = 0.96, *SE* = 0.22, *p* < 0.001, or in Northern Italy, β = 2.05, *SE* = 0.32, *p* < 0.001, were both more likely to belong to the low resilience profile as compared to the high resilience profile relative to adolescents from the German-speaking part of Switzerland. Moreover, students from the Italian-speaking part of Switzerland, β = 0.71, *SE* = 0.15, *p* < 0.001, and Northern Italy, β = 1.83, *SE* = 0.25, *p* < 0.001, versus the German-speaking part of Switzerland had a higher likelihood of being in the average resilience profile as compared to the high resilience profile. In terms of relative risk, adolescents from the Italian-speaking part of Switzerland were about three times more likely (*exp(*β) = 2.61) to belong to the low resilience profile and about two times more likely (*exp(*β) = 2.03) to belong to the average resilience profile as compared to the high resilience profile relative to German-speaking Swiss adolescents. Adolescents from Northern Italy were even at a higher risk, with having an around eight times higher likelihood (*exp(*β) = 7.81) of being in the low and approximately a six times higher risk (*exp(*β) = 6.22) of being in the average resilience profile relative to the high resilience profile when comparing with students from the German-speaking part of Switzerland. When changing the reference category in order to compare differences in terms of risk between the Italian-speaking part of Switzerland and Northern Italy, results revealed that adolescents living in Northern Italy were around three times more likely to belong to the low resilience profile, β = 1.10, *exp(*β) = 2.99, *SE* = 0.29, *p* < 0.001, or the average resilience profile, β = 1.12, *exp(*β) = 3.07, *SE* = 0.25, *p* < 0.001, compared to the high profile. These findings support our hypothesis, suggesting that a higher exposure in the Italian-speaking part of Switzerland and Northern Italy may have been associated with adolescents’ risk of low resilience.

## Discussion

Based on a resilience framework, the current study investigated adolescents’ mental health during the school closures in the beginning of the COVID-19 pandemic as an indicator for adolescents’ healthy functioning during times of crisis. We specifically focused on the role of adolescents’ uncertainties and concerns regarding their education as well as their own and others’ health as risk factors for adolescents’ mental health. Regarding protective factors for adolescents’ mental health, we investigated the relative role of perceived social support by teachers, family, and friends. Based on a person-centered approach, we identified three different resilience profiles, characterized by qualitative and quantitative differences between educational and health concerns, perceived social support by teachers, family, and friends, and well-being and depressive mood. In addition, the findings from a variable-centered approach revealed that educational concerns were a stronger predictor for mental health than health concerns and pointed to a higher relative importance of perceived family support for adolescents’ mental health relative to perceived teacher and friend support.

Lastly, the region in which adolescents lived significantly predicted the likelihood of belonging to one of the profiles: Students from regions being more exposed to the COVID-19 pandemic and related imposed measures (i.e., Italian-speaking part of Switzerland and Northern Italy) were more likely classified in the low or the average rather than in the high resilience profile as compared to students from regions with lower exposure (i.e., German-speaking part of Switzerland).

### Adolescents’ Resilience Profiles

The current study identified three resilience profiles, with 19% of the sample belonging to the low, 47% to the average, and 34% to the high resilience profile. As expected, adolescents categorized in the high resilience profile expressed fewer uncertainties and stress regarding educational demands and high perceived support from teachers, parents, and friends. Also, they reported high levels of well-being and few depressive symptoms. However, compared to those belonging to the average or low resilience profile, adolescents who were part of the high resilience profile did not have fewer worries about others’ health, such as concerns about their families and people around the globe.

These findings are in line with previous research highlighting that educational concerns, such as being worried of not understanding the subject matter, may pose a risk for adolescents’ well-being (Xiang et al., 2019) and that increasing stress associated with new educational challenges during the pandemic predicted lower mental health (Lee, 2020; Stefaniak et al., 2021). Moreover, previous research emphasized the importance of social support for adolescents, especially in times of difficulty or crisis (Armstrong et al., 2005; Wang and Holcombe, 2010). Our findings align with this previous work suggesting that high perceived social support was a particularly valuable resource for adolescent during the school closures related to the COVID-19 pandemic, which highlights the importance of supportive relationship systems during uncertain and difficult times.

The current study also identified a group of adolescents with low resilience, which made up around one fifth of adolescents of the current sample. This group was characterized by high educational concerns, and thus, high uncertainty and stress regarding educational demands during the school closures, while health concerns were at an average level. Recent work demonstrated that adolescents suffered from school closures during the pandemic because their daily routine was highly influenced by changing circumstances regarding their relationships with friends and teachers, losing important anchors in their life (Lee, 2020). Adolescents belonging to the low resilience profile were also those with the relatively lowest perceived social support, which can be perceived as concerning, given that those adolescents would have needed the feeling of being safe and cared the most (Andrews et al., 2002). Future research may identify further risk factors of young people who are particularly vulnerable to educational concerns, as targeting those who are least able to cope with extraordinary situations causing stress would be essential. As the findings of this study were limited due to the cross-sectional design, future work should consider whether pre-existing worries related to school made adolescents more vulnerable during the pandemic-related school closures. For example, the study of Nazar et al. (2020) shows that school burnout was associated with negative mental health.

### The Relative Importance of Educational and Health Concerns for Adolescents’ Resilience

Qualitative differences in adolescents’ resilience profiles emerged regarding the two components of concerns under consideration: educational and health concerns. Previous research showed negative effects on psychological outcomes of the fear of being infected or infecting others (e.g., Brooks et al., 2020). However, in the current study, health concerns of the low resilience profile were at a similar level in the average and the high resilience profile. Moreover, the results from the path analysis revealed that educational concerns were a stronger predictor for mental health than health concerns. Hence, findings suggest that health-related concerns about the pandemic could be factors that are less salient for adolescents as compared to sources of educational concerns.

Literature shows that adolescents already have to cope with a lot of educational stressors independent of a crisis, whereby educational stress has generally been associated with the risk of school burnout and exhaustion at school (Bask and Salmela-Aro, 2013; Nazar et al., 2020; Özhan and Yüksel, 2021) being associated with lower academic achievement (Madigan and Curran, 2021). During the pandemic, adolescents had to deal with many uncertainties about their academic future, potentially increasing this risk for exhaustion. Accordingly, Wang et al. (2020) argued that the educational stress caused by the pandemic might have exceeded adolescents’ resources. In addition, it is also possible that adolescents might have been overwhelmed with distance learning, as the new settings required adaptations and brought new challenges such as technology-based learning (Bond, 2020). Future research may address this topic and investigate whether distance learning with its new technologies may be a factor increasing students’ workload and school burnout.

When considering the descriptive statistics adolescents reported higher health concerns than educational concerns. Hence, adolescents were not without worries about the spread and negative consequences of COVID-19 for others’ health. Moreover, health concerns were indeed negatively related to adolescents’ mental health in the path analysis. This finding is in line with previous studies concluding that adolescents’ mental health (Meherali et al., 2021) or quality of life (Ravens-Sieberer et al., 2021) declined because of COVID-19-related health concerns.

Interestingly, the descriptive findings of the current study also suggest that having more health concerns was positively associated with higher perceived social support in all three sources. Since family interactions were particularly high during the lockdown, high perceived family support might have implied discussing concerns of COVID-19 and the lockdown more strongly within the family. Therefore, adolescents with higher support might have been more aware of risk factors regarding the pandemic resulting in more health concerns. In line with this, Peplak et al. (2021) found child-parent conversations about COVID-19 to be positively correlated with empathic concerns for those affected by COVID-19. Future research may further shed light on the associations between the type of family discussions about COVID-19 and adolescents’ well-being.

### The Relative Importance of Different Forms of Social Support for Adolescents’ Resilience

The three components of perceived social support (i.e., perceived teacher, family, and friend support) had different configurations within the three resilience profiles. First, the profile analyses revealed that perceived teacher and family support differed more between each profile, while perceived friend support varied the least, but was still associated with adolescents’ mental health. In addition, the results from the path analysis revealed that perceived family support had a significantly higher association with adolescents’ mental health than teacher and friend support. Thereby, perceived family support was positively related to both, higher current well-being and lower depressive mood, while teacher and friend support was only significantly and positively associated with current well-being. Hence, for adolescents with higher depressive mood, teacher and friend support did not serve as a protective factor. Lastly, the path analysis only revealed direct effects of perceived social support on mental health. No effects were found with perceived social support as a potential buffer against the negative effects of educational and health concerns on mental health.

The current study included two measures of mental health, one capturing adolescents’ well-being specifically during the time of school closure (i.e., situational measure) and one capturing their depressive mood reflecting a more stable measure, whereby adolescents reported whether they tended to be in certain states over a longer period of time. The idea was that adolescents with higher depressive mood may have had lower resources to deal with the challenges related to the pandemic (Becker and Gregory, 2020; Guessoum et al., 2020; van Loon et al., 2021). Thus, considering potential differences between adolescents’ current well-being and depressive mood, teachers and friends may have helped adolescents to deal better with the current situation, but their perceived support was unrelated to adolescents’ depressive symptomatic. In contrast, adolescents who perceived high family support were more likely to report higher well-being during the school closure and lower depressive mood. While the current study did not assess adolescents’ mental health before the pandemic and thus, cannot make any claims regarding causal relations between support and adolescents’ mental health due to its cross-sectional design, the findings still point to relative differences regarding the importance of the source of which support was received from.

One possible interpretation for the significant role of family support for adolescents’ mental health could be that school closures may have led to a higher amount of time spent with the family. This more intense contact may have increased the need for and importance of family support (Prime et al., 2020). Accordingly, in times of the pandemic, higher parental support was found to be connected to lower anxiety and depression (Klootwijk et al., 2021) as well as lower stress and higher well-being (Guessoum et al., 2020). Pre-pandemic literature points out to direct effects of perceived family support on adolescents’ current well-being and depressive mood. Thereby, Colarossi and Eccles (2003) highlight the importance of perceived mother support on adolescents’ depression and found that effects were larger as compared to perceived friend and teacher support. The authors argue that family support may have cumulative effects over time as parent-child relationships are generally rather long-standing and relatively stable, having notable effects on depression (Garnefski and Diekstra, 1996; Colarossi and Eccles, 2003).

With regards to educational challenges, research showed that parents had to take on new unfamiliar roles in home schooling during the COVID-19 school closures, while being more responsible for the educational support of their children (e.g., Garrote et al., 2021). Regarding educational concerns in the current study, perceived family support was higher in the resilience profile of adolescents with lower educational concerns and vice versa. Moreover, at a general level, parental support correlated with lower educational concerns. However, in the path analysis, perceived family support did not moderate the association between adolescents’ educational nor health concerns and their mental health. Potentially, efforts by parents may not have been sufficient to alleviate the negative association between stress related to educational challenges and adolescents’ mental health. For example, some parents reported being overwhelmed and having problems to motivate their children to study (Garbe et al., 2020). Garrote et al. (2021) found a negative association between stress in parents and their children’s online learning experience. Taken together, parents did play a key role for adolescents’ mental health; however, their role may have been more significant with regards to other functions, such as providing emotional security (Colarossi and Eccles, 2003) or providing a daily routine (Bülow et al., 2021; Liu et al., 2021).

In addition to parents, teachers had an important function to support adolescents in their distance learning during the COVID-19 pandemic. Teachers had to change teaching routines by shifting from face-to-face to online lessons. Therefore, students might have been more dependent on their teachers’ support to guide them through the transition and help them learn in the changed school context. Recent research found high support from teachers and a positive student-teacher relationship to be associated with higher engagement in remote learning during school closures (Bray et al., 2021) as well as fewer mental health problems (Garrote et al., 2021; Ye et al., 2021). Our findings align with this prior work; however, only with regards to adolescents’ current well-being. Moreover, while teacher support did correlate with lower levels of educational concerns, there was no significant moderation effect of teacher support regarding adolescents’ mental health. Potentially, through online teaching, emotional support by teachers might have been more challenging to build up because, among other things, teachers had to deal with new teaching methods. In addition, studies have reported that especially adolescents with mental health issues and special educational needs suffered from school closures with the loss of a daily routine (Lee, 2020). Hence, teachers may have needed additional resources to support at-risk students during the pandemic.

When studying the resilience profiles and particularly the level of perceived support from friends, it was the lowest in the low resilience profile while in the average and the high resilience profile this component was at a similar level. Thus, adolescents being in the low resilience profile felt more isolated during the pandemic, meeting fewer or having fewer friends. It was also reported that this feeling of social disconnection related to higher levels of anxiety and depression as well as lower life satisfaction (Magson et al., 2020). The findings from the variable-centered approach revealed that friend support was positively associated with adolescents’ current well-being, but not with their depressive mood. In line with these findings, Colarossi and Eccles (2003) showed that perceived friend support was only a significant predictor of adolescents’ self-esteem but not of the level of depression. In addition, changes from in-person to online conversations may have been related to loneliness (Rumas et al., 2021). Ellis et al. (2020) suggest that more virtual contact with friends was related to lower loneliness, but also higher levels of depression in adolescents. The authors thereby argue that in group chats adolescents may encounter social aggression and even cyberbullying (Meter and Bauman, 2015; Ellis et al., 2020) which may lead to increasing interpersonal problems. Lastly, even with close friends, and especially in stressful times and dealing with uncertainties, conversations may not only positively, but also negatively affect adolescents’ mental health, when involving excessive discussions of problems and increasingly focusing on negative emotions (Rose, 2002; Ellis et al., 2020). Research may thus shed more light on the specific factors in online conversations with friends that could protect adolescents’ well-being.

### Regional Differences in the Exposure to COVID-19 and Associated Restrictions

A further aim of this study was to identify whether adolescents’ resilience profiles could be predicted by their exposure to the virus with regards to the measures executed and the case numbers. Given that the measures and case numbers varied substantially between regions (Bundesamt für Gesundheit, 2020; Ministero della Salute, 2021), we expected adolescents from the Italian-speaking part of Switzerland and Northern Italy to be more likely to belong to a lower than to a higher resilience profile as compared to the German-speaking part of Switzerland. As hypothesized, the results showed that students living in the German-speaking part of Switzerland were more likely to be in the high than in the average or low resilience profile as compared to those living in the Italian-speaking part of Switzerland or in Northern Italy. In the latter two regions, exposure to COVID-19 was higher, as indicated by the higher case numbers of infected people and more restrictive measures implemented at different points in time (Bundesamt für Gesundheit, 2020; Ministero della Salute, 2021). In Italy for example, the measures no longer allowed to leave the house and schools were closed earlier than in the German-speaking part of Switzerland (Bundesamt für Gesundheit, 2020; Caffo et al., 2020; Ministero della Salute, 2021). Also, while schools where still closed at the time this survey was conducted, the Swiss federal council had already announced their reopening which could have lowered adolescents’ educational concerns. Thus, how adolescents evaluated the measures taken by the government may reveal more information about regarding their resilience in different regions. Thereby, future work would benefit from a longitudinal investigation of adolescents’ development within different regions in order to shed more light on how different measures taken in different areas (e.g., school closures and their duration) predict adolescents’ resilience during the pandemic.

### Socio-Demographic Differences in Adolescents’ Resilience

In addition to regional differences, gender, grade and migration background were significant predictors of adolescents’ likelihood of being categorized into the resilience profiles. Specifically, female students, those in higher-grade levels, and students with a migration background were more likely to belong to the low or average resilience profiles than to the high resilience profile. These results are consistent with previous work, maintaining that during the COVID-19 pandemic females reported higher health concerns and lower mental health (Liu et al., 2020; Wang et al., 2020; Zhou et al., 2020; Nocentini et al., 2021). Moreover, also in line with previous research, increasing age and migration background was associated with lower mental health (Chu et al., 2010; Klein et al., 2020; Zhou et al., 2020; Nocentini et al., 2021; Ravens-Sieberer et al., 2021) resulting in lower resilience. In many instances, parents with a migration background have high aspirations and high expectations for their children (Fuligni, 1997; Glick and White, 2004) but less resources to support them educationally compared to native-born parents (e.g., having a lower SES and being less educated themselves; OECD, 2006), which may have become more noticeable during the pandemic.

However, it must be noted that in the current study, migration background was included as a dichotomous variable, in which each person who had another nationality than or an additional nationality to the Swiss one (i.e., for the Swiss participants) or the Italian one (i.e., for the Italian participants) was considered to have a migration background. Therefore, no country- or nationality-specific statement is possible. It should also be noted with caution that the proportion of students who reported a migration background was very different between the three regions (see Supplementary Table 1 in the Supplementary Material). Future research should include more differentiated and possibly also multiple measures of adolescents’ SES and migration background in order to compare whether different measures would have an effect on the results.

### Limitations

The current study is limited by the cross-sectional nature of the data. Importantly, we could not account for adolescents’ pre-pandemic levels in the key variables and thus not investigate whether there were changes in adolescents’ concerns, perceived social support or mental health. Prior evidence suggests, for example, that adolescents displaying mental health issues prior to the pandemic might have been more vulnerable to shifts in their routines (Meherali et al., 2021). In addition, pre-pandemic reported emotional distress was associated with emotional distress during the pandemic (Shanahan et al., 2020) and pre-pandemic stress influenced COVID-19 concerns and school concerns (van Loon et al., 2021). Hence, those adolescents experiencing higher stress and lower mental health before the pandemic may have been the most affected by COVID-19-related concerns. However, examining causal relations was not the aim of this cross-sectional study. Future research may need to disentangle whether lacking social support may have made adolescents with high levels of educational concerns more vulnerable for mental health issues or whether adolescents with already existing mental health issues may have perceived fewer social support, higher pressure, and uncertainty to cope with the additional educational uncertainties (Lee, 2020).

Similarly, adolescents’ educational concerns may have been already high before the pandemic. Hence, while our study can shed light on the associations between educational and health concerns as risk, and social support as protective factors with adolescents’ mental health, the findings are limited to adolescents’ perceptions of these aspects during the school closures in the early phases of the pandemic and cannot speak for adaptation processes.

Moreover, different sample sizes and characteristics of the regions might have overrepresented a particular region within the full sample. In particular, the Italian-speaking part of Switzerland (i.e., Ticino) was the largest sub-sample with over 700 participants, followed by the German-speaking part of Switzerland and Northern Italy (i.e., Lombardy). The results might therefore be more representative for the situation in Ticino than for the one in the German-speaking part of Switzerland or in Lombardy. Still, the separate analyses of the regions all revealed similar patterns for the three-profile solution; however, it must be noted that the percentage of adolescents that were categorized into the relatively low, average, or high resilience profiles varied between regions, with the German-Speaking part of Switzerland having the highest percentage of adolescents in the low resilience profile (see Supplementary Figures 2, 3, and 4 in the Supplementary Material). Thus, future research needs to examine additional factors (i.e., resources and risk factors specific to adolescents in these regions), which can explain such regional differences.

## Conclusion

The current study provided new insights regarding adolescents’ concerns, perceived support, and mental health during the school closures in early phases of the COVID-19-pandemic. Findings revealed differences between three groups of adolescents, in which different associations between educational concerns, the level of social support by teachers, family, and friends, and their mental health were identified. About one fifth of the sample faced high uncertainty about their educational outcomes and did not feel supported by their environment, pointing to a particularly vulnerable group that may benefit from targeted interventions during school closures. In addition, adolescents being more exposed to COVID-19-related measures and case numbers (i.e., whether they lived in the German-speaking part of Switzerland, the Italian-speaking part of Switzerland or Northern Italy) were at a higher risk of showing rather low resilience. Importantly, our findings also point to a high protective role by adolescents’ family environment during the pandemic.

## Data Availability Statement

The raw data supporting the conclusions of this article will be made available by the authors, without undue reservation.

## Ethics Statement

The studies involving human participants were reviewed and approved by the Ethics Committee of the University of Zurich (Approval N. 20.4.2). Written informed consent from the participants’ legal guardian/next of kin was not required to participate in this study in accordance with the national legislation and the institutional requirements.

## Author Contributions

LD, IB, PC, FL, and JG conceptualized, prepared and realized the study together, including the recruitment of participating schools, data collection, organization, and analysis. LD and JG conducted the statistical analyses of the data and their visualization. IB, PC, LD, and FL provided feedback for the interpretation of the findings, drafted the manuscript, and revised it based on feedback regarding intellectual content from MB and JG. All authors contributed to the article and approved the submitted version.

## Conflict of Interest

The authors declare that the research was conducted in the absence of any commercial or financial relationships that could be construed as a potential conflict of interest.

## Publisher’s Note

All claims expressed in this article are solely those of the authors and do not necessarily represent those of their affiliated organizations, or those of the publisher, the editors and the reviewers. Any product that may be evaluated in this article, or claim that may be made by its manufacturer, is not guaranteed or endorsed by the publisher.
