# Supplementary material for: Educational Concerns, Health Concerns and Mental Health During Early COVID-19 School Closures: The Role of Perceived Support by Teachers, Family, and Friends
Source: Front Psychol. 2022 Jan 25;12:733683. doi: 10.3389/fpsyg.2021.733683 (PMC8821661; doi:10.3389/fpsyg.2021.733683)
Supplement: Supplementary file 1 [file Data_Sheet_1.docx]

Supplementary Material

# Supplementary Tables and Figures

**1.1 Supplementary Tables**

**Supplementary Table 1: Demographic Information**

For details about the demographic information for the total sample as well as for the specific regions are shown in Supplementary Table 1.

**Supplementary Table 2: Full list of measures**

A full list of all measures of the resilience profiles is displayed in Supplementary Table 2, including all items of the components.

## 1.2 Supplementary Figures

**Supplementary Figure 1: Two-Profiles Solution**

The solution with two resilience profiles (Supplementary Figure 1) varied from the three-profile solution in that there was no line (see blue and red line in Supplementary Figure 1) representing average resilience. The high resilience profile was characterized by low levels of *educational concerns* and average levels of *health concerns*, high levels of *perceived social support* (high levels of *perceived teacher* and *family support* and average levels of *perceived friend support*) and high levels of *mental health*. Vice versa, the low resilience profile showed higher levels of *educational* but average levels of *health concerns*, lower levels of *perceived social support* (lower levels of *perceived teacher* and *family support* and average levels of *perceived friend support*) and low levels of *mental health*. However, both high and low resilience profiles showed quite similar patterns as compared to the three-profile solution. In the two-profile solution, 50% were classified in the low resilience profile and 50% were characterized by the high resilience profile.

**Supplementary Figures 2, 3 and 4: Three-Profiles Solution Validation**

To validate the three resilience profiles identified for the entire sample, we replicated the latent profile analysis for the three investigated regions. The three-profile solution of the German-speaking part of Switzerland (*N* = 486; 69% females; age: *M* = 15.80, *SD* = 1.31, range = 14-20) is displayed in Supplementary Figure 2. In this solution, 38% were assigned to the low resilience profile, 43% to the average one, and 19% were characterized by the high resilience profile. The three-profiles solution of the Italian-speaking part of Switzerland (*N* = 760; 64% females; age: *M* = 16.30, *SD* = 1.66, range = 14-20) is displayed in Supplementary Figure 3. Here, 33% of the adolescents were identified to belong the low resilience profile, 50% to the average one and 17% to the high resilience profile. Northern Italy (*N* = 316; 93% females; age: *M* = 16.45, *SD* = 1.17, range = 15-19) is displayed in Supplementary Figure 4. This solution revealed that 12% of the adolescents belonged to the low resilience profile, 63% to the average one and 25% to the high resilience profile.

When inspecting the three Figures, the patterns of the results are very similar to the three-profile solution with the entire sample. The high resilience profile was characterized by low levels of *educational concerns*, average levels of *health concerns*, high *perceived teacher, family* and *friend support* and high levels of *mental health* indicators. The average resilience profile showed average levels in all components. The low resilience profile showed an inverse configuration to the high resilience profile except that levels of *health concerns* were rather low and *perceived friend support* was somewhat higher compared to *perceived teacher* and *family support* in the low resilience profile. Only small deviations from the solution of the entire sample were noticeable. For example, in Northern Italy, *health concerns* of the low resilience profile were at the lowest levels among all profiles and in the German-speaking part of Switzerland *health concerns* were on the same level in all three resilience profiles. Moreover, in the Italian-speaking part of Switzerland *perceived friend support* had a somewhat higher level for the average than for the high resilience profile, which wasn’t the case in the entire sample as well as in the German-speaking part of Switzerland and in Northern Italy (in the entire sample the high resilience profile had around the same level of *perceived friend support* compared to the average profile). However, despite these small differences the main pattern of the three-profile solution in the entire sample could be replicated in all regions.

|  |  |  |  | Regions | | |
| --- | --- | --- | --- | --- | --- | --- |
|  | Total sample  *N =* 1562 |  |  | German-speaking part of Switzerland  *N =* 486 | Italian-speaking part of Switzerland  *N =* 760 | Northern Italy  *N =* 316 |
| Age (years)  Range | 16.18 (SD = 1.48)  14-20 |  |  | 15.80 (SD = 1.31)  14-20 | 16.30 (SD = 1.66)  14-20 | 16.48 (SD = 1.17)  15-19 |
| Gender (% female) | 72.1 |  |  | 69.1 | 64.4 | 93.1 |
| Grade (%)  7  8  9  10  11  12  13 | 0.7  15.7  20.9  19.5  19.4  17.6  6.2 |  |  | 1.4  32.1  21.8  14.4  15.1  13.9  1.2 | 0.4  9.5  23.5  21.7  16.2  16.3  12.4 | -  -  12.3  23.7  36.1  27.9  - |
| School type (%)  Secondary school  High school  Apprenticeship/vocational school  Middle school  Higher technical school  University / college  Other | 26.2  58.4  12.0  2.6 0.7  -  0.1 |  |  | 38.4  53.2  -  8.4  -  -  - | 30.2  42.3  25.8  -  1.5  -  0.2 | -  100.0  -  -  -  -  - |
| Migration background (%) | 26.9 |  |  | 31.0 | 34.3 | 5.2 |
| SES (% own house) | 75.1 |  |  | 70.4 | 68.7 | 95.2 |

**Supplementary Table 1**

*Demographic information*

*Note.* Gender: 1 = female, 0 = male; SES: 1 = own house, 0 = rented house; Migration background: 1 = migration background, 0 = no migration background.

**Supplementary Table 2**

|  | Items |
| --- | --- |
| Concerns |  |
| (1) Educational concerns | “I feel overwhelmed with school.”; “I am often unmotivated for school.”; “I often feel like I can't make it through school.”; “I am afraid that I will have to repeat the school year.” |
| (2) Health concerns | “I am worried that my friends or my family could become seriously ill because of Corona.”; “I am worried that I could infect my friends or my family with Corona.”; “I am worried that, around the globe, many people will fall sick.”; “I am worried that people will become seriously ill if I don’t follow the rules.”; “I am worried that Corona continues to spread.” |
| Perceived social support |  |
| (3) Perceived teacher support | “My teachers always help me when I get stuck.”; “My teachers notice when I have a problem.”; “My teachers think it is important that I am doing well.” |
| (4) Perceived family support | “I can always rely on my family.”; “When I'm under stress and pressure, I find support from my family.”; “I can tell my family everything.” |
| (5) Perceived friend support | “I can always rely on my friends.”; “When I'm under stress and pressure, I find support from my friends.”; “I can tell my friends everything.” |
| Mental health |  |
| (6) Current well-being | “I felt happy.”; “I felt fit.”; “I was full of energy.” “I did well with the school work.” “I got along well with my family.” |
| (7) Depressive mood* | “I often feel sad or unhappy.”*; “I often feel lonely.”*; “I feel isolated at the moment.”* |

*Complete List of Items of the Components of the Resilience Profiles*

*Note*. Items and scales marked with an asterisk* were recoded in order to build the profiles so that higher values of depressive mood reflect lower depressive levels.


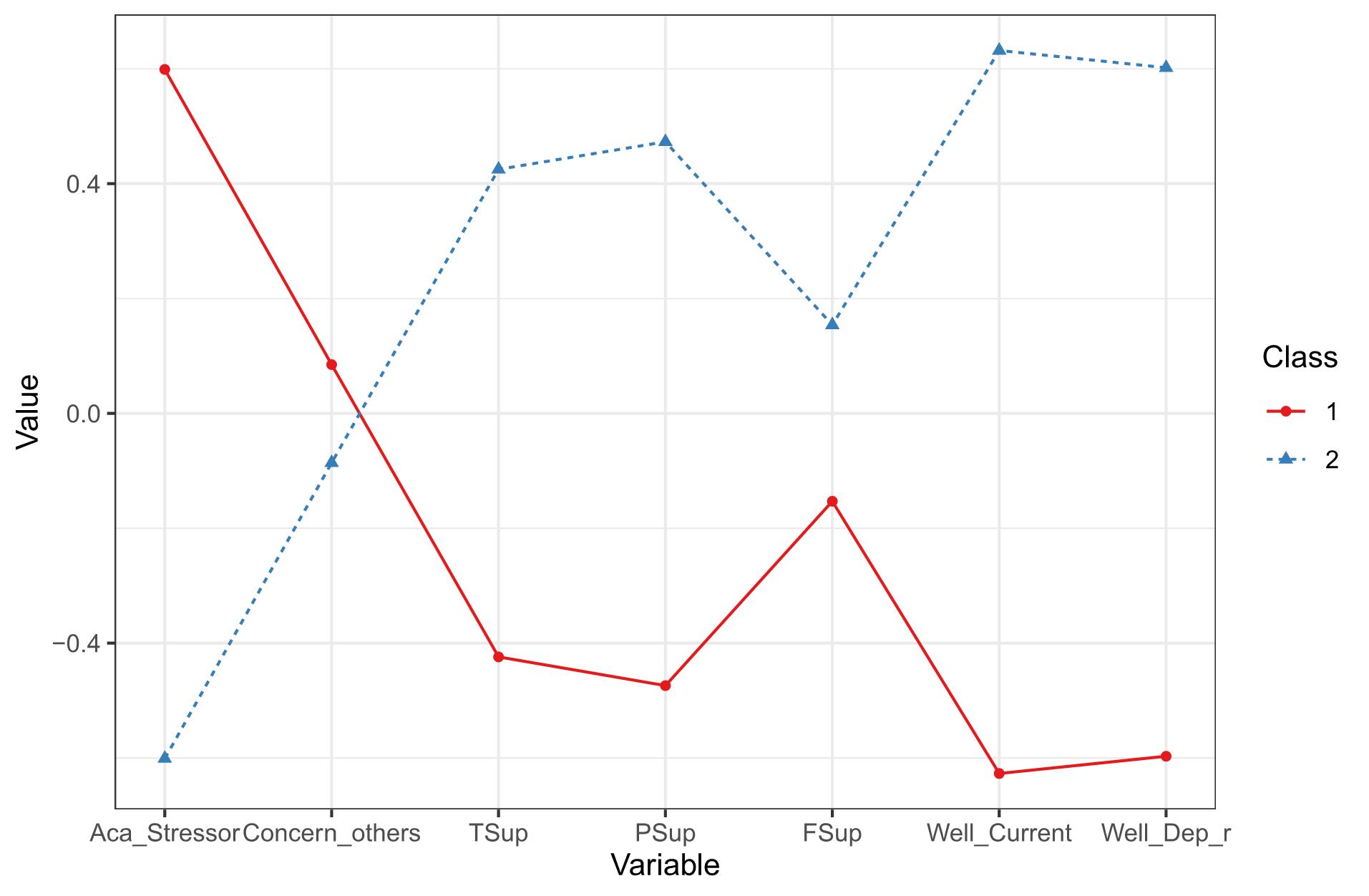


Resilience

profiles

Low (50.20%)

High (49.80%)

Mean +/- 1 SD

Educational concerns

Current well-being

Depressive mood*

Health concerns

Perceived family

support

Perceived friend support

Perceived teacher

support

Components

**Supplementary Figure 1.** Latent resilience profiles: Two-profiles solution. Scales marked with an asterisk* were recoded so that higher values of depressive mood reflect lower depressive levels. All components were mean-centered.


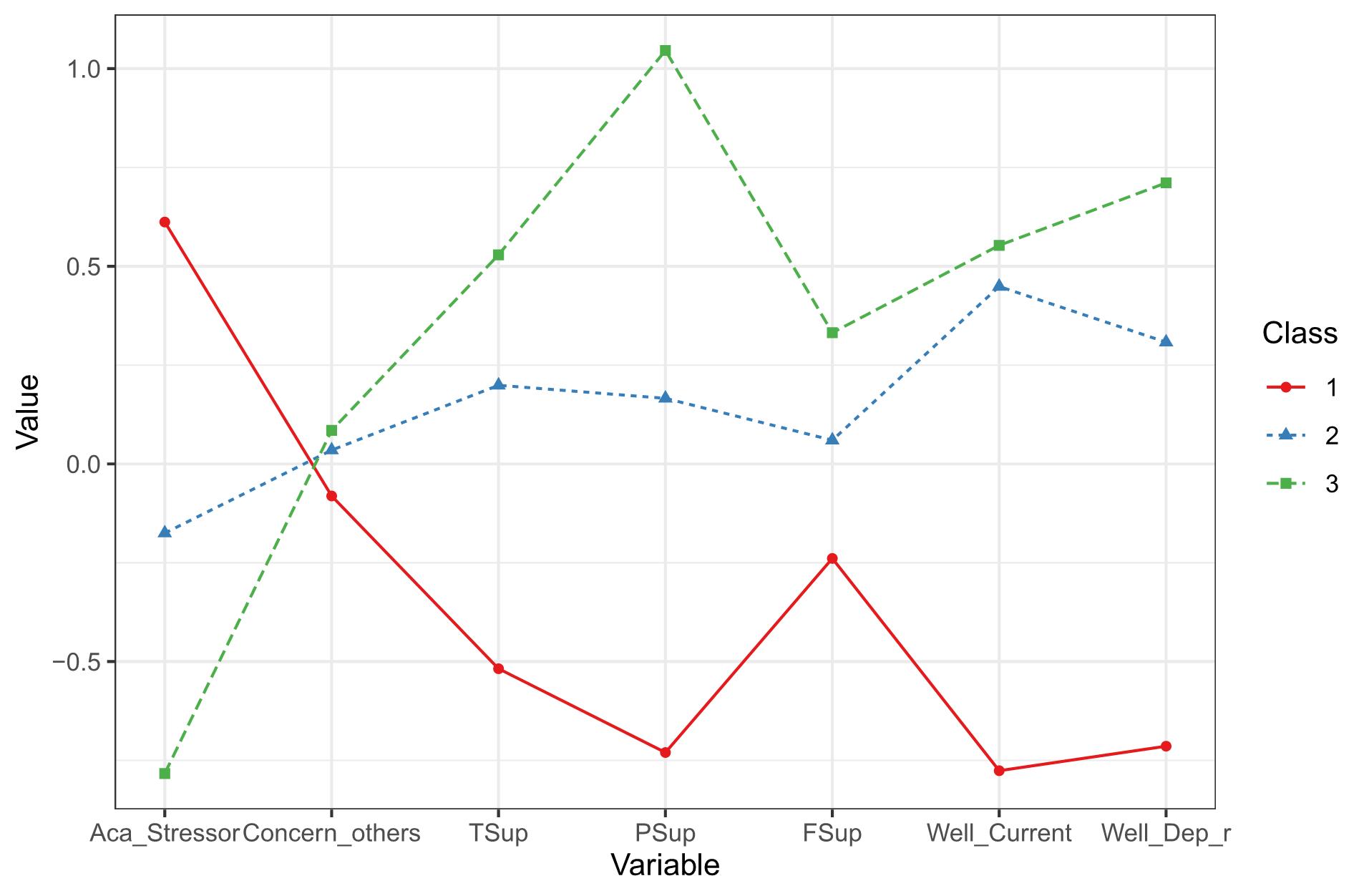


Resilience

profiles

Low (38.26%)

Average (43.13%)

High (18.61%)

Mean +/- 1 SD

Educational concerns

Health concerns

Perceived friend support

Current well-being

Depressive mood*

Perceived family

support

Perceived teacher

support

Components

Components

**Supplementary Figure 2.** Latent resilience profiles: Three-profiles solution of the German-speaking part of Switzerland. Scales marked with an asterisk* were recoded so that higher values of depressive mood reflect lower depressive levels. All components were mean-centered.


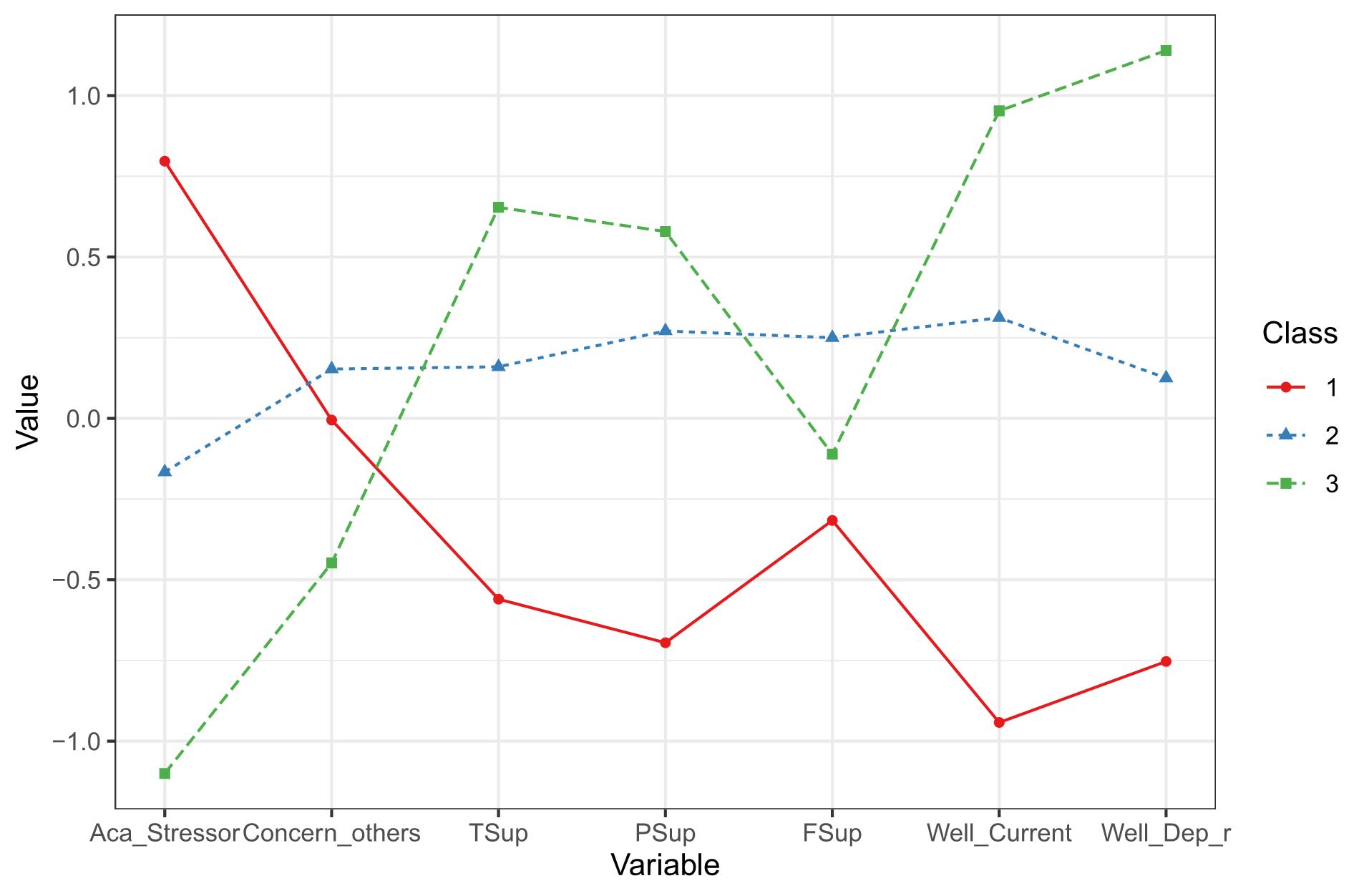


Resilience

profiles

Mean +/- 1 SD

Low (33.42%)

Average (49.84%)

High (16.74%)

Health concerns

Depressive mood*

Current well-being

Perceived friend

support

Perceived family

support

Perceived teacher

support

Educational concerns

Components

**Supplementary Figure 3.** Latent resilience profiles: Three-profiles solution of the Italian-speaking part of Switzerland. Scales marked with an asterisk* were recoded so that higher values of depressive mood reflect lower depressive levels. All components were mean-centered.


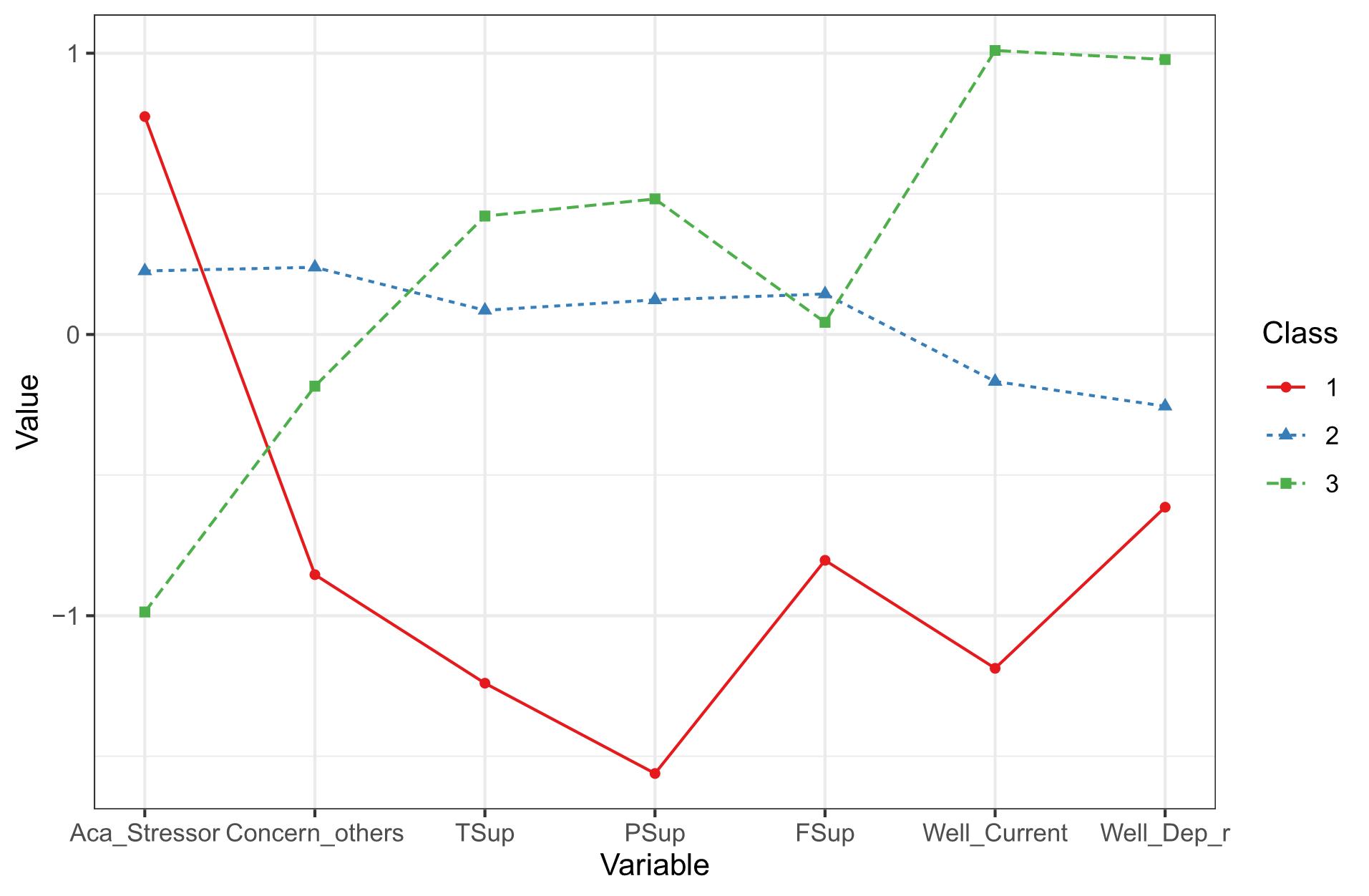


Resilience

profiles

Mean +/- 1 SD

Low (12.11%)

Average (63.20%)

High (24.69%)

Depressive mood*

Current well-being

Perceived friend

support

Perceived family

support

Perceived teacher

support

Health concerns

Educational concerns

Components

**Supplementary Figure 4.** Latent resilience profiles: Three-profiles solution of Northern Italy. Scales marked with an asterisk* were recoded so that higher values of depressive mood reflect lower depressive levels. All components were mean-centered.
